# Wearable-Sensor-Based Physical Activity and Sleep in Children with Down Syndrome Aged 0–5 Years: A Systematic Review

**DOI:** 10.3390/s25237278

**Published:** 2025-11-29

**Authors:** Gilson Borges, Vanessa Moreira, Fabio Bertapelli

**Affiliations:** Department of Human Movement Sciences, Federal University of São Paulo, Santos 11015-020, Brazil; gilson.borges20@gmail.com (G.B.); vanessa.chaves@unifesp.br (V.M.)

**Keywords:** Down syndrome, physical activity, sleep, wearable device, accelerometry, systematic review

## Abstract

Wearables enable objective measurement of physical activity (PA) and sleep. Studies that have examined PA and sleep in children with Down syndrome (DS) have not been systematically reviewed. The objectives of this systematic review (PROSPERO: CRD420251036478) were to: (1) describe patterns of PA and sleep in children with DS; (2) compare PA and sleep between DS and non-DS; and (3) evaluate sensor data collection procedures. Searches were conducted in PubMed, Scopus, Web of Science, Embase, and SPORTDiscus, with the last search on 7 October 2025. Risk of bias was assessed with the Joanna Briggs Institute tools. From 203 records, 9 original studies were included. Children with DS (n = 8–66 participants; 1–67 months) showed small changes in movement rates over time and greater upper- than lower-limb movements. Segment-specific counts and time spent on high-intensity activity were lower in DS than non-DS. Overall, children with DS exhibited poor sleep quality, sleeping approximately one hour less than controls and 3–7 h below global recommendations. Sensor data collection protocols varied in epoch length (15–30 s), attachment site (wrist, ankle, and hip), and device model. Population-based research employing standardized sensor procedures is warranted to better establish PA levels and sleep quality in children with DS.

## 1. Introduction

Adequate physical activity (PA), limited sedentary behavior (SB), and high-quality sleep are essential for motor skill acquisition, social and cognitive development, emotional regulation, adiposity control, and linear growth in early childhood [1,2,3,4,5]. In 2019, the World Health Organization (WHO) published guidelines that indicate PA and sleep are essential components of health and developmental outcomes in children under five years of age [6]. WHO recommends that infants (i.e., 0 to 11.9 months) engage in interactive floor-based play and accumulate 30 min of tummy time daily, while toddlers (i.e., 12 to 35.9 months) and preschoolers (i.e., 36 to 59.9 months) should achieve at least 180 min of PA. WHO guidelines also provide strong recommendations on good-quality sleep, including naps, that should range from 12 to 17 h for infants, 11–14 for toddlers, and 10–13 for preschoolers.

Wearable devices are important for the objective monitoring of PA and sleep. These are miniaturized, lightweight, high-tech systems worn directly on the skin or attached to the trunk or limbs through bands (e.g., wristbands), belts (e.g., waist belt), accessory-based platforms (e.g., watches), textiles (e.g., sensor-integrated garments), and adhesive patches [7,8]. Modern devices embed multiple sensors, such as accelerometers and gyroscopes, which, through advanced signal analysis and machine learning functionalities, allow for accurate measurement of continuous movement behavior [9,10], serving as a complement to direct observation of PA and polysomnographic-derived sleep [11,12]. Accuracy of wearable sensors is supported by extensive evidence addressing placement, sampling rate, epoch length, filtering options, non-wear detection, and algorithms for activity and sleep classification [13,14]. As demonstrated in two comprehensive reviews by Lettink et al. [14] and Schoch et al. [13], the Actiwatch 2 sensor remains the most frequently used device for sleep assessment, whereas ActiGraph sensors continue to dominate physical activity measurement due to their extensive validation and widespread adoption across childhood movement studies. In the pediatric population, comprehensive reviews have demonstrated wearable capabilities in measuring general movements, PA type classification and intensity spectrum, and sleep–wake states in children aged 0–5 years [14,15]. Reviews have further demonstrated that wearable technology is essential for PA and sleep surveillance in individuals with metabolic and pulmonary diseases [16], mobility impairments [17], and autism spectrum disorder [18]. Importantly, global recommendations have been established for the use of wearables for the monitoring of PA and sleep in both research and clinical practice [19,20]. Nonetheless, evidence synthesis remains limited in young children with disabilities.

Down syndrome (DS) is a genomic disorder caused by trisomy of chromosome 21 [21]. Prevalence estimates of live births with DS range from 1:600 to 1:900 across low-, middle-, and high-income countries [22,23,24]. Individuals with DS commonly present with significant limitations in body functions and structures, including muscle hypotonia, congenital heart defects, hearing and vision impairments, endocrinological and gastrointestinal disorders, and intellectual disability [25]. Additionally, a recent scientific statement from the American Heart Association indicates two other factors that impact health among individuals with DS from birth: PA and sleep [26]. Some systematic reviews have addressed patterns of PA, SB, and sleep in DS. Agiovlasitis et al. [27] observed that wearable-based mean sedentary time across studies was 552 min/day in individuals with DS. Notably, among the seventeen studies reviewed, one study [28] demonstrated that moderate-to-vigorous PA from direct observation was consistently low in children with DS aged 3–5 years. Another systematic review of eight studies reported wearable-based PA levels ranging from 0% to 43% in individuals with DS under 22 years of age [29]. However, this review [29] identified only one study that examined PA and sleep in young children (i.e., 3–6 months) with DS, showing reduced leg activity and poor-quality sleep [30]. However, there is no review that provides a comprehensive and systematic synthesis of wearable-based PA and sleep in children with DS aged 0–5 years.

The purpose of this systematic review is threefold: (1) to examine patterns of wearable-based PA and sleep in children with DS aged 0–5 years; (2) to compare PA and sleep outcomes between children with DS and without DS; and (3) to evaluate wearable-based data collection methods—such as sampling rates, epoch lengths, attachment sites, and device models—as well as analytical approaches for assessing PA and sleep.

## 2. Materials and Methods

### 2.1. Eligibility Criteria

Studies were included if they met the following criteria: (1) enrolled children with DS aged 0 to 71.9 months; (2) were original peer-reviewed articles published in English; (3) were cohort, cross-sectional, case–control, randomized clinical trials, and quasi-experimental studies; and (4) assessed PA or sleep using wearable devices. Studies were excluded if they included children with DS whose data were not analyzed separately from other groups. Reviews, opinions, books, book chapters, editorials, commentaries, protocols, case reports, diagnostic, and methodological and qualitative studies were also excluded.

### 2.2. Information Sources

The literature search was conducted in five databases: PubMed, Scopus, Web of Science, Embase, and SPORTDiscus. No restrictions were applied regarding the year of publication. In addition to database searches, citation searching was performed manually through reference list checking. Dissertations, conference abstracts, and unpublished reports were not considered in the present review. The last search was conducted on 7 October 2025.

The search strategy integrated controlled vocabulary from the Medical Subject Headings (MeSH) and free-text keywords from wearable-based PA and sleep studies. In addition, the “Glossary of Terms” from the WHO “Guidelines on Physical Activity, Sedentary Behaviour and Sleep” document [6] was also consulted to ensure comprehensive terms. The search terms were structured following population descriptors, constructs of PA and sleep, and the assessment of these constructs. Therefore, the complete set of terms applied was as follows: (a) Down syndrome—Down syndrome, Downs syndrome, and trisomy 21; (b) age group—infan*, child*, toddler*, newborn*, neonate*, baby, babies; (c) physical activity and sleep—physical activit*, physical exercise, fitness, motor skill, energy expenditure, motor development, fundamental movement skill, metabolic equivalent of task, sedentary behavio*, sedentary time, sleep, nap, and naps; and (d) wearable devices—wearable sensor*, wearable device*, electronic device*, acceleromet*, physical activity monitor*, activity tracker, and actigraph*. Truncation symbol (i.e., *) was applied to capture word variants (e.g., infan* retrieved “infant”, “infants”, “infancy”; physical activit* retrieved “physical activity” and “physical activities”; sedentary behavio* retrieved “sedentary behavior” and “sedentary behaviour”; and acceleromet* retrieved “accelerometer”, “accelerometer” and “accelerometry”). The search strategy is presented in Table 1.

### 2.3. Study Selection Process

Before initiating the screening process for the present review, two reviewers completed training sessions covering the PRISMA 2020 guideline [31]. Following training, the two reviewers independently applied the eligibility criteria to a random sample of 20 records to ensure consistent application and interpretation by the reviewers. Inter-rater reliability was almost perfect (κ = 0.888). The study selection process was conducted in three stages. In the first stage, all records retrieved from the databases were imported into the Rayyan platform (https://www.rayyan.ai/, accessed on 5 October 2025). Rayyan is a free, web-based systematic review management platform developed by the Qatar Computing Research Institute to assist researchers in conducting systematic reviews [32]. In Rayyan, duplicate records were automatically identified and then subjected to manual verification to ensure accurate removal. Following deduplication, the titles and abstracts of the remaining records were independently examined by the reviewers for eligibility. Each study was coded as “include”, “exclude”, or “undetermined” and conflicts were flagged within Rayyan. Discrepancies were resolved through discussion and consensus. Inter-reviewer agreement was assessed using Cohen’s Kappa statistics, with values ≥ 0.81 interpreted as indicative of almost perfect agreement [33]. In the second stage, the full texts of potentially eligible studies were retrieved and independently assessed for eligibility by the reviewers. Again, discrepancies between reviewers were resolved through discussion until consensus was achieved. Involvement of a third reviewer was not required. In the third stage, the reference lists of all included studies were manually screened to identify additional records, which were assessed in the same manner.

### 2.4. Data Extraction

Data were independently extracted by the two reviewers. Following the selection process, any discrepancies were resolved through discussion until consensus was achieved. Data were extracted across five aspects: (1) Study characteristics: author, year of publication, country, exclusion criteria, and study design. (2) Participant characteristics: sample size, nationality, chronological age, sex, ethnicity, and gestational age. (3) Device specifications: make and model, sampling rate, measurement axes, epoch length, number of sensors per participant, attachment site, monitoring period, average wear time, and software and algorithms used for analysis. (4) Daily PA outcomes: time in PA, segment-specific movement, leg and trunk activity minutes and counts, counts per minute, and acceleration parameters. In the present review, daily PA was defined as activity measured by wearable devices over 24 h per day. (5) Sleep outcomes: total sleep time, sleep efficiency, sleep fragmentation, wake time, sleep onset latency, number of nocturnal awakenings, wake after sleep onset (WASO), and circadian rhythmicity. Data on the characteristics of participants without DS from case–control studies were also extracted. Statistical information for group comparisons, such as *p* values, was extracted whenever reported. For the extraction of data related to PA and sleep outcomes, reviewers followed the description of accelerometer-based descriptors provided by the GRANADA consensus [19]. In cases where quantitative data were not available in tables or text, the WebPlotDigitizer tool—Version 5.2 was used to retrieve values from figures [34,35].

### 2.5. Risk of Bias Assessment

The risk of bias was assessed using the Joanna Briggs Institute (JBI) critical appraisal checklists [36]. A checklist was selected according to the study design (cross-sectional, cohort, randomized controlled trial, and quasi-experimental). The two reviewers independently conducted the appraisal, and disagreements were resolved through discussion until consensus was reached; no automation tools were employed in this process. Each checklist item was rated as “Yes” (scored as 1), “No”, “Unclear”, or “Not applicable” (scored as 0). To allow comparability across studies, a percentage score was calculated as the number of “Yes” responses divided by the total number of applicable items, multiplied by 100. Items rated as “Not applicable” were excluded from the denominator. Studies were classified as having low risk of bias (≥75% “Yes”), moderate risk (50–74% “Yes”), or high risk (<50% “Yes”). Following the JBI guidelines, we further conducted domain-specific judgments. We critically examined five bias domains: (i) participant selection and sampling; (ii) exposure and outcome measurement validity; (iii) confounding control; (iv) completeness of follow-up and adherence; and (v) blinding, where applicable. Reviewer discussions focused on methodological rigor and contextual relevance of each domain, rather than only on checklist completion.

### 2.6. Synthesis Methods

Statistics (means, standard deviations, and *p* values) were extracted and presented in text, tables, and figures. Mean total sleep time for children with and without DS was displayed alongside the WHO’s minimum recommended sleep duration for each corresponding age range using a radar chart generated in Python version 3.12.12. For this purpose, we converted the mean total sleep time from minutes to hours in two studies [37,38].

Meta-analysis was not feasible due to heterogeneity in outcomes and measurement protocols across studies. To ensure comparability across studies, age categories were harmonized according to the WHO Guidelines [6]: infants (0–11.9 months), toddlers (12–35.9 months), and preschoolers (36–59.9 months).

### 2.7. Protocol and Registration

We developed this systematic review in accordance with the Preferred Reporting Items for Systematic Reviews and Meta-Analyses (PRISMA) 2020 statement [31]. The review was registered in the International prospective register of systematic reviews of the National Institute for Health and Care Research (PROSPERO; registration number: CRD420251036478). The full review protocol is available as a PDF format in the PROSPERO database: https://www.crd.york.ac.uk/PROSPERO/view/CRD420251036478, accessed on 1 October 2025.

## 3. Results

### 3.1. Study Selection

**Figure 1:**
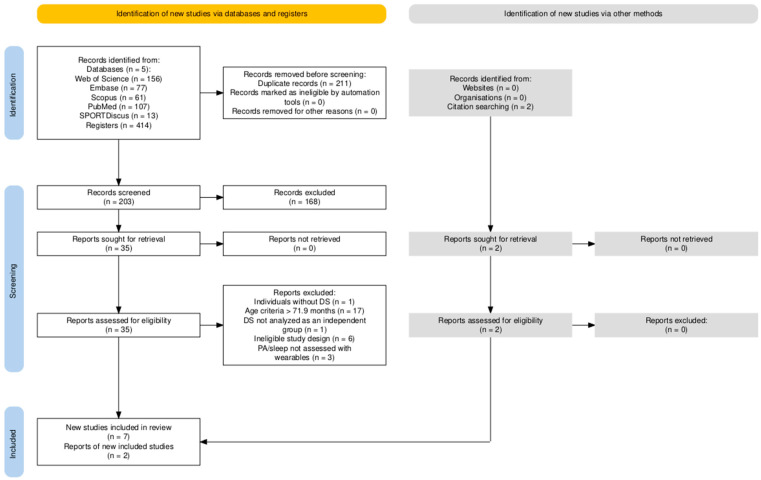
Flow diagram of systematic search and screening of studies.

A total of 414 records were identified through the database searches, and two additional records were identified through reference list screening. After removing 211 duplicates, 203 records were screened through title and abstract examination, of which 168 were excluded. Of the 35 full-text studies assessed for eligibility, 28 were excluded due to the following reasons: individuals without DS (n = 1); age > 71.9 months (n = 17); studies that included children with DS but did not report or analyze their data separately from other groups (n = 1); non-eligible study design (n = 6); and not assessing PA or sleep with wearable devices (n = 3). A total of two records identified through reference list screening were eligible and included. In total, nine studies met the inclusion criteria and were included in the review. Agreement between the two independent reviewers was almost perfect (κ = 0.886). The study selection process is summarized in the PRISMA flow diagram (Figure 1).

### 3.2. Risk of Bias

Results of the assessment of risk of bias are presented in Table 2. All four cross-sectional studies [37,38,39,40] met 100% of the criteria and were classified as having a low risk of bias. Two cohort studies [30,41] and one quasi-experimental study [42] fulfilled more than 80% of the evaluated items, being similarly classified as low risk. Two additional studies, one cohort [43] and one randomized clinical trial [44], met 54.55% and 61.54%, respectively, of the criteria and were therefore considered to have a moderate risk of bias. No study was classified as high risk. Domain-level analysis indicated that most studies clearly described inclusion criteria, outcome measurement, and statistical analysis. Common weaknesses occurred in cohort and randomized designs, particularly incomplete follow-up and absence of blinding. No study showed systematic measurement bias in wearable-device outcomes. Because seven studies were classified as low risk and two as moderate, the overall certainty of the synthesized evidence was considered acceptable; however, conclusions from the two moderate-risk studies were interpreted cautiously.

At the item level, the most frequently unmet criteria were related to adequate follow-up in cohort studies and the identification and management of potential confounding factors. In the randomized clinical trial, the main limitations concerned the absence of blinding of participants and treatment providers, as well as insufficient clarity regarding whether outcome assessors were blinded. The predominance of studies classified as low risk of bias supports the overall reliability of the evidence base.

### 3.3. Study Characteristics

#### 3.3.1. Country, Period, and Study Design

Most of the studies (89%) were conducted in the United States of America. The studies were published between 2006 and 2021 and employed four designs (i.e., cross-sectional, four studies; longitudinal, three studies; randomized clinical trial, one study; and quasi-experimental, one study).

#### 3.3.2. Participant Characteristics

Characteristics of participants are presented in Table 3. Studies included individuals with DS (nine studies; n = 8–66 participants) and typical development children (TD) as comparison groups (seven studies; n = 8–43 participants). The chronological ages reported in the studies were as follows: DS samples (nine studies, age range: 1–67 months; mean age range = 3.3–51.8 months); TD samples: (seven studies, age range: 1–58 months; and mean age range = 3.3–48.3 months).

Based on WHO age definitions for children under five years [6], three studies investigated infants (0–11.9 months; [30,40,42]), whereas the remaining studies included toddlers (12–35.9 months) or preschoolers (> 36 months). Only three studies [40,42,43] stratified analyses by age subgroups. None of the studies included samples covering the entire 0–5-year age span. Gestational age was reported in one study (gestational age: 36 weeks). Three studies reported the ethnicity of children with DS, including African American (n = 5), Caucasian (n = 61), European American (n = 7), Hispanic/Latino (n = 1), and other ethnicities (n = 6). No study reported the nationality of participants. Studies reported exclusion criteria related to the diagnosis of DS (one study, i.e., mosaicism or translocation), gestational age (two studies, i.e., <36 weeks), medical conditions (four studies, e.g., seizure disorders, vision problems, heart defects, and other impairments), language barriers (one study, i.e., non-English-speaking parents), and protocol adherence (two studies, i.e., valid days of actigraphy data). A total of three studies did not report any exclusion criteria.

Regarding confirmation of DS status, three studies [37,38,41] reported genetic verification by karyotype. Lloyd et al. [41] identified 28 participants with free trisomy 21 and 2 with mosaicism, while Fernandez et al. [38] confirmed diagnosis through genetic testing at enrollment. The remaining studies identified participants based on clinical diagnosis or recruitment through DS clinics and early-intervention programs, without specifying whether karyotype confirmation was performed. None of the included studies relied solely on parental report.

In accordance with the JBI critical appraisal, we extracted whether each study reported a statement on the ethical approval process. Although PRISMA guidelines do not recommend extraction or reporting ethical approval process, JBI emphasizes ethical transparency as part of study quality. Several included studies stated institutional review board or ethics committee approval and written parental consent prior to data collection [30,37,38,39,40,41,42,43,44] whereas others [30,40]—although conducted in academic or clinical settings—did not specify the approval body or consent process.

Cross-cultural and socioeconomic differences should be considered when interpreting the generalizability of findings. Socioeconomic and parental education characteristics were described in four studies. Edgin et al. [37] reported that 40% of families had annual household incomes below USD 40,000 and that most mothers had completed at least some college or university education. Hauck et al. [43] indicated that families were primarily from middle-income households (USD 60,000–80,000 per year) and that most mothers had a higher university education (n = 7). Arias-Trejo et al. [39] presented measures of family income in DS (i.e., USD 11,600–USD 34,999) and maternal education (i.e., undergraduate degree). The remaining studies did not report socioeconomic or parental education information. Among the included studies, most reported comparable baseline characteristics between children with DS and TD peers. McKay et al. [30] was the only study that observed statistically significant baseline differences, specifically in anthropometric measures such as length (i.e., lower in DS vs. TD) and skinfold measures (i.e., higher in DS vs. TD). Three studies [41,42,43] did not report statistics regarding demographic differences between DS and TD children. The remaining studies [37,38,39,40,44] reported that no significant differences were observed between groups for age, sex, or socioeconomic indicators.

### 3.4. Outcomes

Of the nine included studies, five investigated exclusively on PA [40,41,42,43,44], three focused exclusively on sleep [37,38,39], and one examined both PA and sleep outcomes [30]. All studies examined PA and sleep in free-living conditions [38,39,40,42,43]. No studies conducted data collection in laboratory settings.

### 3.5. Measurement Protocols

#### 3.5.1. PA and Sleep Sensor Configuration

**Figure 2:**
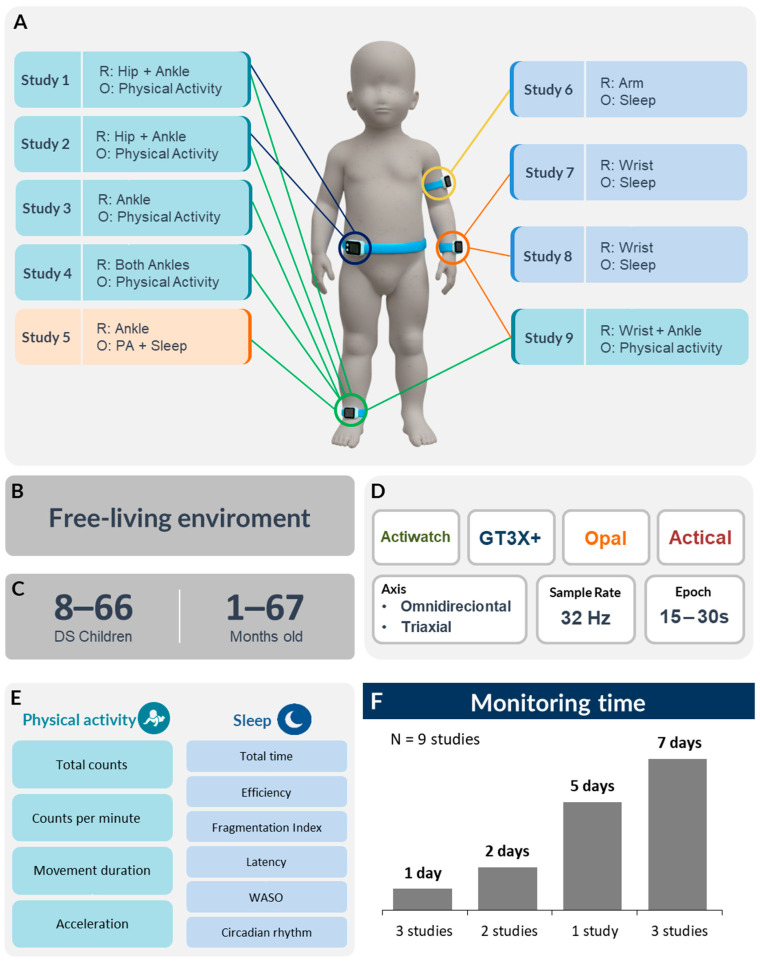
Methodological characteristics of sensor-based studies on PA and sleep in DS. Note: (**A**) attachment sites and outcomes; (**B**) monitoring environment; (**C**) sample characteristics (sample size and age); (**D**) sensor characteristics (sensor model, axis, sampling rate, and epoch length); (**E**) physical activity and sleep outcomes; (**F**) sensor monitoring time; DS = Down syndrome; PA = physical activity; WASO = wake after sleep onset; R = region; O = outcome; Hz = Hertz; Study 1 = [44]; Study 2 = [41]; Study 3 = [43]; Study 4 = [42]; Study 5 = [30]; Study 6 = [39]; Study 7 = [37]; Study 8 = [38]; Study 9 = [40]. Color lines indicate sensors placed on the wrist (orange), ankle (green), hip (blue), and arm (yellow).

All studies relied on actigraphy-based devices (GT3X+, Actiwatch models, Philips Respironics or Mini Mitter, and the Opal movement sensor) and assessed PA and sleep. Devices were worn on combined hip/ankle, unilateral arm, wrist, or ankle in sleep studies, and on the bilateral or unilateral ankle, and unilateral combined hip and ankle. Epoch lengths ranged from 15 to 30 s and a sampling rate of 32 Hz was used. The sensor technical specifications are presented in Table 4. Monitoring protocols involved continuous 24 h recordings for five days (one study), seven days (three studies), a day (wake hours, one study), 24 h (three studies), and 48 h (one study). PA and sleep sensor configuration are shown in Figure 2.

#### 3.5.2. Data Processing and Algorithms

##### Non-Wear Periods, Device Removal, and Missing Data

Across the included studies, procedures for handling non-wear periods, device removal, and missing data varied considerably. Several studies reported explicit criteria for identifying and excluding non-wear periods. Ketcheson et al. [40] removed intervals associated with bathing, swimming, or device loss, based on detailed parental logs that were cross-checked with raw accelerometer data. This study also implemented a zero-value threshold, excluding “periods with at least two consecutive minutes of zeros” from wear-time calculations. Hauck et al. [43] similarly relied on parental logs to record device removal and cross-validated these reports with raw accelerometer counts to identify movements not attributable to the infant. Other studies established inclusion thresholds based on the duration of valid data. Fernandez et al. [38] required at least five consecutive days of valid actigraphy, excluding 14 participants who did not meet this criterion. Arias-Trejo et al. [39] applied comparable criteria (minimum of five days) and additionally excluded data from periods coinciding with major life events (e.g., family travel, bereavement, or social/cultural activities) likely to disrupt sleep or activity patterns. Several studies provided only limited methodological detail. McKay et al. [30] removed “induced movement” events noted in parental logs but acknowledged technical limitations due to battery failures and incomplete records following artifact removal. Lloyd et al. [41] reported removing data with zero values but did not specify how device removal was identified. Angulo-Barroso et al. [44] applied a similar approach, excluding “epoch values equal to zero” without documentation of removal criteria. Finally, Edgin et al. [37] used parental sleep logs to examine discrepancies between actigraphy-derived and parent-reported sleep variables.

##### Software, Algorithm, and Classification Criteria

Software: In sleep studies, data were processed using Actiware software [37,38,39] and Mini Mitter proprietary algorithms [30], while PA studies processed the data with Actilife, Mini Mitter/Respironics software, and MATLAB. Analysis parameters are presented in Table 5.

Algorithm: For PA studies, algorithms were not required, as these investigations did not classify participants into specific PA categories. For sleep assessment, only one study (Fernandez et al.) [38] reported the algorithm used for sleep analysis (i.e., template matching algorithm).

Classification criteria: McKay et al. [30] described a multi-step analytical process that involved (1) detection of individual day–night transitions, (2) separation of high- and low-intensity activity, and (3) classification of sleep–wake states using a manufacturer-defined sensitivity setting (threshold = 80). Fernandez et al. [38] and Edgin et al. [37] applied a medium-sensitivity threshold (40 counts per epoch or per minute) to assess sleep variables, while Arias-Trejo et al. [39] used the same threshold, correcting sleep onset and offset manually with sleep logs. Hauck et al. [43] relied on logbooks to exclude artifact (e.g., handling by an adult or a motorized infant device) periods. Khasgiwale et al. [42] used MATLAB for wearable-sensor data analysis. 

### 3.6. Summary of Findings

#### 3.6.1. Physical Activity

A summary of findings is presented in Table 6. There was marked heterogeneity in PA outcomes across the six included studies. Two studies focused on segment-specific movement (i.e., leg and trunk activity minutes and counts; [41,44]), two reported counts per minute (CPM) at different sensor sites (i.e., wrist vs. ankle; [40,43]), two provided longitudinal or age-based trajectories of counts [30,43], and one examined acceleration parameters (average and peak acceleration, movements per hour, and seconds per movement; [42]).

Overall, studies that examined PA in children with DS aged 0–5 years observed (1) within-group site effects on CPM, with higher values at the wrist than ankle (n = 1 study), (2) lower PA levels in DS than TD at matched ages (n = 4 studies), and (3) attenuated or plateaued PA trajectories across infancy (n = 2 studies). Significant differences were observed between wrist- and ankle-derived CPM in children with DS. Ketcheson et al. [40] reported that, in children with DS, wrist sensors captured higher PA levels (mean CPM = 469.75) than ankle sensors (mean CPM = 332.98). With respect to PA trajectories, studies observed both total counts and segment-specific movement patterns. Ketcheson et al. [40] showed that infants with DS began the first two months with a higher wrist activity than TD children (406.69 vs. 353.89 counts), peaked at 9–10 months (599.33 counts), and then declined by the end of the first year, whereas TD infants increased steadily to 705.30 counts at 11–12 months. A similar pattern was observed at the ankle, where DS infants demonstrated an early rise followed by a plateau, while TD infants displayed a sustained increase across age intervals. Hauck et al. [43] extended these observations to 18 months, reporting modest CPM increases in DS (49.16 at 1 month to 131.48 at 18 months) compared with a steeper trajectory in TD (61.99 to 264.54 CPM). McKay et al. [30] further confirmed group differences between 3 and 6 months, showing that infants with DS had significantly more low-intensity leg movements and fewer high-intensity movements than TD peers, with differences becoming more pronounced by 6 months (*p* < 0.05).

With respect to segment-specific patterns, data from Lloyd et al. [41] demonstrated that leg high-activity movements in DS infants remained relatively stable between 10 and 14 months (45,382 to 50,123 units), while leg low-activity movements fluctuated modestly (21,810 to 23,030 units). In contrast, trunk activity increased more substantially, with high-intensity movements rising from 10,296 to 16,392 units and low-intensity movements growing from 8805 to over 9000.

Intervention studies were reported in two studies. Angulo-Barroso et al. [44] examined leg and trunk activity during a treadmill training program and observed that infants in the high-intensity group showed greater increases in high-activity movements and spent less time in low activity than those in the low-intensity group (*p* < 0.05), with these differences persisting up to 15 months after walking onset. Khasgiwale et al. [42] examined the effects of an in-home kicking intervention in infants with DS aged 3–5 months. Following eight weeks of training, the DS group increased their mean leg movement rate from 2002 to 2351 movements/hour of awake time (*p* < 0.05), but values remained below those of TD peers (3344 movements/hour). No significant changes were observed in mean or peak acceleration or in movement duration from pre- to post-intervention.

Taken together, these findings indicate that although PA in children with DS has been examined using different approaches, the studies demonstrate attenuated or plateaued activity trajectories and lower intensity compared with TD peers, with some evidence that targeted interventions may enhance activity levels.

#### 3.6.2. Sleep

**Figure 3:**
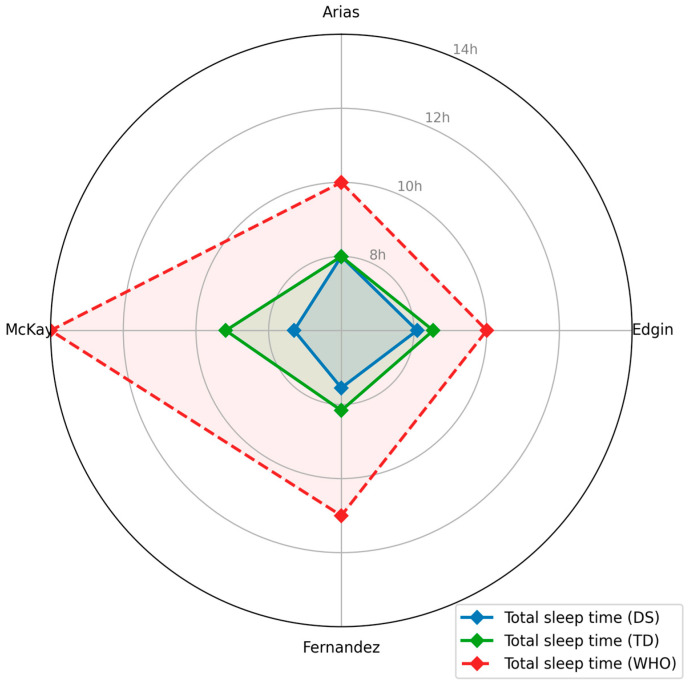
Radar chart of total sleep time across studies in children with Down syndrome (DS), typical development (TD) controls, and World Health Organization (WHO) [6] recommendations. Note: Blue, green, and red lines show total sleep time data for Down syndrome (mean hours), typical development children (mean hours), and World Health Organization (total minimum recommended hours), respectively. DS = Down syndrome; TD = typical development children; Arias = [39]; Edgin = [37]; Fernandez = [38]; McKay = [30].

A summary of findings is presented in Table 6*,* Figure 2 and Figure 3. Four studies examined sleep in children with DS. All studies examined total sleep time [30,37,38,39]; three examined sleep efficiency and sleep onset latency [37,38,39]; two examined fragmentation index, minutes of wake time after sleep onset (WASO) [37,39]; one examined circadian rhythm parameters and sleep–wake fragmentation [38]; and one evaluated sleep longitudinally [30]. In summary, the sleep studies indicated (a) reduced total sleep time and sleep efficiency in children with DS, (b) increased sleep onset and sleep fragmentation, and (c) altered circadian rhythm profiles compared with TD peers.

Across studies [30,37,38,39], total sleep time was significantly shorter in DS (7.3–8.5 h/night) compared with TD peers (8.2–9.2 h/night). Additionally, Figure 3 presents the comparison of total sleep time (hours) between children with DS and TD peers across studies, relative to the minimum total sleep time (hours) recommended by the WHO [6] for corresponding age groups. Figure 3 shows that, across all studies, children with DS and TD consistently slept fewer hours, remaining below the WHO recommended sleep range for each age group, with the greatest deficit observed at ages 3 and 6 months. Sleep efficiency was also significantly reduced in DS (DS: 74 to 84%; TD: 83 to 89%). Measures of night fragmentation were reported in three studies [30,37,39], all showing greater sleep disruption in DS. Finally, Fernandez et al. [38] found that the development of circadian rhythmicity in young children with DS paralleled that of TD peers. Mean interdaily stability (0.455 vs. 0.435) and mean intradaily variability (0.729 vs. 0.725) did not differ between groups. Likewise, circadian amplitude analyses showed comparable rhythmic strength between groups. In contrast, children with DS had lower mean relative amplitude (DS: 0.888; TD: 0.935), higher mean nighttime activity during the least active period (DS: 29.32; and TD: 17.34), while daytime activity during the most active hours was similar.

Taken together, these findings indicate that children with DS had shorter total sleep time, lower efficient sleep, increased fragmentation, and normal circadian disruption compared with their TD peers.

## 4. Discussion

This systematic review synthesized evidence from nine studies investigating PA and sleep using wearable devices in children with DS aged 0–5 years. Overall, the findings indicate that children with DS exhibit lower PA levels, earlier plateaus in PA trajectories, and fewer high-intensity movements compared with TD peers. In relation to sleep, the reviewed studies consistently reported shorter total sleep time, lower sleep efficiency, and greater night fragmentation among children with DS. The studies also revealed variations in wearable-based data collection methods, including differences in device models, epoch lengths, attachment sites, and monitoring duration.

### 4.1. Physical Activity and Sleep Findings

According to the World Health Organization [6], children under five years of age should engage in regular PA, limit sedentary behavior, and obtain sufficient, good-quality sleep. The studies included in this review demonstrate that children with DS engage in less PA and experience poorer sleep quality compared with TD children. Disparities should be addressed through early-life interventions that promote movement opportunities. In this regard, two PA intervention studies included in this review, treadmill training [44] and an in-home kicking intervention [42], showed increases in activity levels, suggesting that early interventions can help mitigate inactivity in infants with DS, though their outcomes remained below those observed in TD children.

Our synthesis of total sleep time across studies reveals a consistent pattern of reduced sleep duration among children with DS relative to both TD children and WHO child recommendations [6]. Across studies [30,37,38,39], total sleep time ranged between 7 and 8 h in DS groups, compared with 8 to 9 h in TD children, falling below the minimum recommended by WHO for this developmental period, 10–14 h. Evidence from polysomnography studies in younger populations with DS support these findings. Heubi et al. [45] reported a mean sleep duration of 7 h in children with DS, while Kolstad et al. [46] observed a mean of 8 h in preschoolers. Although polysomnographic data in children with DS remains limited, these findings suggest a consistent pattern of shortened total sleep duration across measurement methods and early developmental stages. This highlights that wearable devices may offer a reliable low-cost approach to extend sleep research in DS, particularly during developmental periods when polysomnography is less feasible. Nonetheless, validation of sensor-derived sleep estimated against polysomnography in infancy is warranted to establish their accuracy and clinical applicability in this population.

The current findings also align with previous systematic reviews conducted in older children with DS, which consistently reported low PA levels and high sedentary time [27,29]. Importantly, evidence from reviews involving other developmental and functional disabilities—such as mobility impairments [17], autism spectrum disorder [18], and chronic metabolic or pulmonary diseases [16]—also supports the feasibility and clinical relevance of wearable-based monitoring for health surveillance.

### 4.2. PA and Sleep Sensor Settings

The assessment of PA and sleep in infants, toddlers, and preschoolers relied primarily on accelerometer-based sensors with ActiGraph GT3X+, Actiwatch, and Respironics Actical models. Only one study [42] used the Opals sensor that embedded multiple sensors including an accelerometer, gyroscope, and magnetometer. Previous studies demonstrated that these device models provided feasible and valid objective measurements of movement and rest patterns in free-living environments [3,15,42,47,48].

For PA examination, most studies relied on mean counts per minute as the primary outcome. However, raw acceleration data were analyzed with monitoring durations ranging between 24 h (three studies), 48 h (two studies), and 7 days (one study). A previous study in the general pediatric population using the GT3X model found that infants who were not walking achieved reliable activity estimates with only one day of accelerometer data [49], indicating that the monitoring duration employed in the reviewed studies (i.e., ≥24 h) is consistent with previous research. However, the reliability of actigraphy when compared across different monitoring durations (hours or days) has not yet been investigated in infants with DS. Regarding body placement, most PA studies positioned the devices on the unilateral or bilateral ankle (three studies), followed by combined hip/ankle (two studies), and combined wrist/ankle (one study). In toddlers and preschoolers from the general population, both hip and wrist placements yield valid estimates of total PA [15].

Regarding sampling rate, four of the six included studies reported a sampling rate of 32 Hz. The remaining studies—Arias et al. [39] and Edgin et al. [37] (Actiwatch sensors), Hauck et al. [43] (Actical sensor), Ketcheson et al. [40] (GT3X sensor), and Khasgiwale et al. [42] (Opal sensor)—did not provide this information. Nevertheless, based on manufacturer specifications (Table 4), the nominal sampling rates of these devices are 32 Hz for Actiwatch and Respironics Actical, 30–100 Hz for the ActiGraph GT3X, and 1280 Hz for the Opal sensor. While these values allow an approximate comparison across studies, the precise sampling rate used in the study by Ketcheson et al. [40] remains indeterminate, as the exact configuration within the 30–100 Hz range is not specified. It is important to note that, as highlighted in a previous review [50], data collection in most accelerometer firmware is optimized for 30 Hz. Therefore, sampling frequencies that are multiples of 30 (e.g., 30 or 60 Hz) tend to yield more accurate and comparable estimates of movement across devices. With respect to epoch length, most PA studies included in our review used 15 s epochs, which aligns with the recommendation of using epochs shorter than 60 s to adequately capture the sporadic and intermittent nature of physical behaviors in young children [15]. All devices used in the included studies contained multidirectional sensors, which is relevant because they capture acceleration across multiple directions, providing a more comprehensive measure of total movement.

Information on sensitivity and filtering was inconsistently presented, often with incomplete technical detail. Fernandez et al. and Edgin et al. applied medium-sensitivity thresholds (40 counts per epoch or minute), while Arias-Trejo et al. [39] used similar criteria with sleep-log correction. Moreover, investigations did not specify filtering parameters. Evidence from adults with DS suggests that filtering plays an important role in accurately capturing low-intensity movements—a feature particularly relevant given the hypotonia and reduced acceleration amplitude characteristics of this population. Bertapelli et al. [51] demonstrated that enabling the low-frequency extension (LFE) filter in ActiGraph accelerometers reduced step-counting error from approximately 31% to 10% in adults with DS. In another study, Bertapelli et al. [52] showed that applying the LFE configuration improved the prediction of oxygen uptake from step counts. Despite these findings, there is no evidence regarding the use of low-frequency or other filters designed to enhance signal processing to small and slow movements in infants with DS. Considering that infants with DS exhibit lower activity counts than TD peers, the absence of optimized filtering may contribute to underestimation of true movement levels. Future research should systematically evaluate the effect of filtering parameters on accelerometer accuracy in infants with DS to improve low-amplitude motor behavior.

Another concern relates to the sleep-scoring algorithms used to estimate sleep in DS. None of the studies specified which algorithm was applied to derive sleep–wake outcomes such as total sleep time or sleep efficiency; only software was reported. This is a critical issue, as estimates of sleep outcomes in infants are highly dependent on the selected algorithm. In a comprehensive review by Schoch et al. [13], 47.5% of studies did not report algorithms for actigraphy-based sleep estimates in infants. Additionally, the review [13] observed that algorithms varied substantially depending on proprietary algorithms, software updates, and device-specific implementations, many of which are not disclosed to users. Although the four studies in our review reported the device (Respironics) and did not specify the algorithm applied, previous evidence indicates that the Respironics algorithm demonstrates acceptable accuracy (77–84%) for sleep–wake classification in infants without disabilities [53]. However, to our knowledge, no studies have validated the performance of this device specifically in children with DS. Because commercial actigraphy systems frequently offer multiple or evolving algorithms that are not always validated for infants, such a lack of methodological detail limits reproducibility and comparability across studies. Future research should report algorithm specifications to ensure greater transparency and interpretability of sleep-related findings.

Regarding sleep research, most studies in this review examined total sleep time, sleep efficiency, sleep onset latency, and number of nocturnal awakenings. The American Academy of Sleep Medicine (AASM) Clinical Practice Guideline [54] substantiates the methodological choices commonly adopted in research with young children. The guideline recommends actigraphy as an objective tool to estimate sleep parameters, including total sleep time. However, a review has advised caution when using actigraphy to estimate wake after sleep onset [47]. Importantly, the AASM guideline recommends a minimum monitoring duration of 72 h for both clinical and research applications, which aligns with the 5–7 consecutive days of monitoring adopted in most studies reviewed [37,38,39], except for one study [30] that used a 48 h protocol. Regarding device placement, one study positioned the device on the arm, two studies on the wrist, and one on the ankle. The sleep guideline acknowledges that actigraphy devices can be worn on the wrist, ankle, or waist, depending on feasibility and study objectives [55]. Regarding epoch length, two studies used 15 s epochs [30,39], while two employed 30 s intervals [37,38]. As polysomnography—the criterion measure—typically uses 60 s epochs, caution is warranted when interpreting actigraphy–PSG agreement based on shorter epochs. As emphasized by Sadeh [13], the evaluation of actigraphy validity should consider the high sensitivity in epoch-by-epoch PSG–actigraphy comparisons.

No study included in this review examined potential reactivity to the devices. Qualitative evidence from past research [49] indicated that wearable sensors were generally well tolerated by infants, with mild irritability or discomfort typically limited to the first day of use. Caregivers reported occasional difficulties with clothing fit and concerns about tightness or skin irritation, particularly for ankle placement. These findings emphasize the importance of considering device size, placement, and monitoring duration to ensure comfort and minimize interference with infants’ normal routines. Therefore, future studies involving infants with DS should account for possible reactivity on the first day of monitoring and consider excluding this initial period from data processing to improve measurement accuracy.

Another methodological aspect relates to the handling of non-wear periods, device removal, and missing data. Although several studies implemented parental logs, zero-value thresholds, or validity criteria (e.g., minimum days of data) to identify and exclude non-wear time, the approaches varied widely. The absence of standardized definitions and processing rules for valid wear time limits comparability across studies and may introduce bias in estimates of total activity and sleep duration. Future research should examine wear-time validation and missing-data handling to enhance the reliability and reproducibility of actigraphy-based assessments in early childhood. The U.S. National Health and Nutrition Examination Survey (NHANES) defines valid accelerometer data as recordings with at least 10 h of wear time per day for a minimum of four days. In infants, however, there are no standardized, sensor-based guidelines for handling missing data. A protocol study conducted in infants of the general population reported that invalid data segments were imputed based on time-matched averages from other valid days, offering a systematic approach to preserve data continuity and reduce bias associated with brief periods of missing or corrupted recordings [49].

### 4.3. Quality of Research

Across the nine included studies, overall risk of bias was predominantly low (seven studies). No studies were judged as high risk. For sleep, quality was consistently strong, yielding a clear low-risk profile (100% rated as “yes”). In a recent pediatric clinical sleep review of inpatient samples of children [55], we identified seven observational actigraphy studies that used similar JBI appraisal checklists. Based on our calculations using Hybsch supplementary tables from Mann et al. [55], JBI weight ranged from 33 to 89%, with five studies classified as moderate risk of bias, one as high, and one as low. With respect to PA, however, studies in our review showed greater variability: of six studies, four (67%) were low risk and two (33%) moderate, with none at high risk. Where present, elevated risk primarily reflected incomplete adjustment for confounding and the practical challenges of blinding. Collectively, these findings indicate a consistent methodological strength in the DS sleep literature, contrasted with a more heterogeneous quality profile for PA studies. According to JBI principles, considering the relative weight of evidence by the risk-of-bias domain enhances the credibility of synthesis. Given that seven of the nine studies were rated as low risk, we have reasonable confidence in the direction of the observed effects. Nevertheless, some uncertainty remains in domains related to confounding and attrition, which may influence the magnitude of the observed differences between DS and non-DS groups.

### 4.4. Limitations of the Evidence

While these findings help clarify early sleep and activity profiles in children with DS, our synthesis also underscores an important limitation related to substantial heterogeneity across studies, particularly in how PA was measured, processed, and reported, which affects the comparability of results. Considerable variability was observed in PA outcomes, ranging from CPM and movement bouts to raw acceleration features with cross-sectional, RCT, quasi-experimental, and longitudinal designs. For example, Hauck et al. [43] used an ankle-mounted Actical device over a 24 h protocol and reported mean CPM of 49–104 in children with DS aged 1–12 months, whereas Ketcheson et al. [40], using a wrist- and ankle-mounted ActiGraph GT3X over seven consecutive days, found higher CPM values (189–460 at the wrist and 354–705 at the ankle) in children with DS aged 1–12 months. Such discrepancies likely reflect differences in device specificities, placement sites, and data-processing algorithms rather than true biological variation. Therefore, between-study comparisons of CPM values should be interpreted cautiously.

To address these challenges and enhance cross-study comparability, recent initiatives, most notably the GRANADA consensus [19], have provided detailed recommendations for the standardization of accelerometer data processing and analysis of sleep and PA. The consensus encourages a shift from traditional count-based metrics toward more robust analytical frameworks such as compositional data analysis and machine learning-based models. It further emphasizes the derivation of accelerometer data descriptors of interest, including time-use behavior (e.g., time in PA intensities), average acceleration (e.g., measured in counts per day or mg), and scalar descriptors (e.g., bouts, breaks, variability, and sleep efficiency) to improve reproducibility and facilitate meta-analytic integration. Adopting these standardized procedures in future research involving young children with DS would support the development of evidence-based recommendations for movement behaviors for early-life movement and sleep behaviors in this population.

Beyond methodological heterogeneity, analytical approaches to examine changes in PA and sleep over time varied across studies. Although three studies [30,40,43] employed longitudinal or repeated-measures approaches, each presented methodological differences that limited interpretation of PA change over time. Ref [30] used monthly follow-ups from 3 to 6 months, whereas Ketcheson et al. [40] analyzed bimonthly cohorts from 1 to 12 months cross-sectionally, and Hauck et al. [43] followed infants up to 18 months with sparse measurement intervals. No studies applied growth curve or nonlinear modeling capable of quantifying PA and sleep changes. Moreover, small DS samples (n = 8–11) and differences in sensor sites (wrist vs. ankle) hindered comparability and may have obscured subtle maturational patterns. Notably, no studies to date have examined longitudinal changes in sleep parameters in this age group (0–5 years), leaving developmental patterns of sleep duration largely unexplored. Future research should employ multi-wave accelerometry and modeling approaches to examine trajectories of both PA and sleep.

An additional limitation of the evidence relates to group comparability across essential baseline characteristics. Most studies matched or statistically controlled for age and sex between children with DS and TD peers, minimizing demographic bias. However, some studies inconsistently reported information on socioeconomic status, parental education, and coexisting medical conditions. Only a few studies excluded participants with medical conditions, while others did not specify exclusion criteria for comorbidities. It is also important to note that the relatively small sample sizes of the studies may partly explain the lack of such analyses. Furthermore, evidence on PA and sleep derives predominantly from studies conducted in high-income countries (i.e., the United States). Such context is not representative of the broader DS population in low- and middle-income countries (LMICs). Children from LMICs are more likely to experience differences in PA opportunities and access to health-promoting resources [53]. Taken together, observed between-group differences in PA or sleep outcomes should be interpreted with caution, as they may partly reflect unmeasured influences related to socioeconomic or clinical conditions. Moreover, there is an urgent need for research conducted in LMICs to better capture the contextual determinants of these outcomes and enhance the generalizability of findings across diverse populations of infants with DS.

Regarding the diagnosis of trisomy 21 and its potential impact on outcomes, two studies included participants with mosaicism (n = 2, Lloyd et al. [41]) or translocation (n = 1, Fernandez et al. [38]), indicating limited representation of DS types within the reviewed literature. DS type may contribute to differences in health, motor, and cognitive development, as previous studies have shown that individuals with mosaicism or translocation often exhibit milder phenotypic characteristics than those with free trisomy 21 [56]. Nevertheless, given the low prevalence of mosaicism and translocation (approximately 2–5% of all DS cases), most studies remain limited to examining associations of DS variations with PA or sleep outcomes. Future research should report chromosomal variations and consider analytic approaches to clarify whether variations within DS contribute to PA and sleep trajectories.

### 4.5. Limitations of the Review

Although the present review employed a comprehensive search across several databases and rigorous dual screening with high inter-reviewer agreement, some limitations warrant consideration. First, restriction to English-language publications may have introduced language bias. Second, data extraction relied on what was reported in the articles, and, in some cases, on values derived from figures. Finally, methodological heterogeneity across studies precludes meta-analysis. In PA research, only two of the six studies provided counts per minute, and even these differed by device family (Actical vs. ActiGraph), body placement (ankle vs. wrist + ankle), and monitoring duration (24 h vs. 7 days). Other studies reported incomparable outcomes such as movement counts, accelerations, or movement rates. As a result, any pooled estimate would reflect device and analytic differences rather than true PA behavior. For sleep outcomes, similar inconsistencies in monitoring time, epoch length, and sensor placement hindered cross-study standardization. Future research adopting harmonized protocols and reporting of raw acceleration data—as encouraged by the GRANADA consensus—will be critical to enable valid meta-analytic synthesis. Because meta-analysis was not feasible, small-study effects could not be quantified. In addition, a small number of eligible studies precluded assessment of publication bias using, for instance, funnel plots or regression tests. Nevertheless, we acknowledge the possibility of selective publication favoring studies with significant or interpretable results, which may overestimate between-group differences. This limitation is noted when interpreting the conclusions of lower PA and poor sleep in DS.

### 4.6. Implications for Research

Although the present review offers an integrative overview of available findings, the evidence base remains limited in scope and statistical power. Most studies included small samples (n = 8 to 66 participants) drawn from high-income countries, limiting generalizability. Geographic imbalance threatens the validity and equity of global recommendations for child health and development [57]. Expanding research in low- and middle-income countries (LMICs) is essential, given the higher prevalence of key exposures and outcomes in these settings—such as all-cause mortality, maternal–child undernutrition, and infectious disease morbidity—and the likelihood that contextual factors (e.g., caregiving practices, built environments, sleep routines, and opportunities for PA) shape 24 h movement behaviors in distinct ways [58]. Prioritizing LMIC cohorts, with locally validated wearables, would be critical to generate recommendations that are both accurate and globally applicable. The variability in PA and sleep parameters across studies further hindered comparability, and only two intervention studies were identified, neither of which included wearable-based sleep outcomes. Additionally, future research should prioritize larger, longitudinal samples; harmonized protocols; and concurrent assessments of PA and sleep to explore bidirectional associations between PA and sleep behaviors during early development.

### 4.7. Implications for Clinical Practice

The findings of this review have important implications for clinical practice and policy. Early detection of insufficient PA and sleep disturbances in DS should be incorporated into pediatric surveillance. Clinicians and therapists may integrate wearable-based monitoring into early intervention programs to track behavioral progress. The American Heart Association’s 2024 Scientific Statement on Health Promotion in DS identified PA and sleep as two fundamental, modifiable determinants of long-term health beginning at birth [26]. Integrating these behaviors into pediatric surveillance may therefore provide a practical pathway to improve health outcomes across the lifespan. Consistent with this framework, wearable-based monitoring could serve as a feasible tool to detect early behavioral risk patterns and guide individualized interventions within primary care settings.

## 5. Conclusions

This systematic review provides the first integrated synthesis of PA and sleep patterns in young children with DS using wearable technologies. The findings indicated that children with DS were at higher risk for physical inactivity and poor sleep quality. Despite generally high methodological quality, substantial heterogeneity in sensor-based protocols and varied study designs limited statistical synthesis. Future studies should prioritize harmonized analytical approaches, longitudinal designs, and concurrent assessment of PA and sleep to capture developmental trajectories more accurately.

## Figures and Tables

**Table 1:** Search strategy.

Keywords Concept	Keywords Domain	Keywords Strategy
Population descriptors	Down syndrome	“Down syndrome” OR “Downs syndrome” OR “trisomy 21”
	AND	
	Age group	Infan * OR child * OR toddler* OR newborn * OR neonate * OR baby OR babies
	AND	
Constructs of physical activity and sleep	Physical activity and sleep	“physical activit *” OR “physical exercise” OR fitness OR “motor skill” OR “energy expenditure” OR “motor development” OR “fundamental movement skill” OR “metabolic equivalent of task” OR “sedentary behavior” OR “sedentary time” OR sleep OR nap OR naps
	AND	
Assessment of physical activity and sleep	Wearable devices	“wearable sensor *” OR “wearable device *” OR “electronic device *” OR acceleromet * OR “physical activity monitor *” OR “activity tracker” OR actigraph *

Note: * = truncation operator; OR, AND = Boolean operators.

**Table 2:** Risk of bias assessment of included studies.

1º Author, Year	Item 1	Item 2	Item 3	Item 4	Item 5	Item 6	Item 7	Item 8	Item 9	Item 10	Item 11	Item 12	Item 13	Weight %	Risk of Bias
**Cross-sectional studies ^a^**															
Arias-Trejo, 2020 [39]	Yes	Yes	Yes	Yes	Yes	Yes	Yes	Yes	-	-	-	-	-	100	Low
Edgin, 2015 [37]	Yes	Yes	Yes	Yes	Yes	Yes	Yes	Yes	-	-	-	-	-	100	Low
Fernandez, 2017 [38]	Yes	Yes	Yes	Yes	Yes	Yes	Yes	Yes	-	-	-	-	-	100	Low
Ketcheson, 2017 [40]	Yes	Yes	Yes	Yes	Yes	Yes	Yes	Yes	-	-	-	-	-	100	Low
**Cohort studies ^b^**															
Hauck, 2020 [43]	Unclear	Yes	Yes	Unclear	Unclear	Yes	Yes	Yes	Unclear	Unclear	Yes	-	-	54.55	Moderate
Lloyd, 2010 [41]	NA	NA	Yes	Yes	Yes	Yes	Yes	Yes	Yes	NA	Yes	-	-	100	Low
McKay, 2006 [30]	Yes	Yes	Yes	Yes	No	Yes	Yes	Yes	Yes	No	Yes	-	-	81.82	Low
**Quasi-experimental studies ^c^**															
Khasgiwale, 2021 [42]	Yes	No	NA	Yes	Yes	Yes	Yes	Yes	Yes	-	-	-	-	87.5	Low
**Randomized clinical trial ^d^**															
Angulo-Barroso, 2008 [44]	Yes	Unclear	Yes	No	No	Yes	Unclear	Yes	Yes	Yes	No	Yes	Yes	61.54	Moderate

Note: NA = not applicable; ^a^ = studies assessed with eight items from the cross-sectional JBI checklist; ^b^ = studies assessed with eleven items from the cohort JBI checklist; ^c^ = studies assessed with nine items from the quasi-experimental JBI checklist; ^d^ = studies assessed with thirteen items from the randomized clinical trial JBI checklist; - = item not available in the checklist.

**Table 3:** Characteristics of included studies.

1º Author, Year	Sample Size, DS	Age in Months Mean (SD)	Ethnicity/Race n (%)	Exclusion Criteria	TD Comparison Group	Study Design	Country
Angulo-Barroso, 2008 [44]	30	21.8 (3.1) and 24.9 (5.1) Age range: NA	African American: 2 (6.7%), Caucasian: 26 (86.7%), Other: 2 (6.6%)	NA	No	RCT	USA
Arias-Trejo, 2020 [39]	18	50.2 (5.2) Age range: 24–60	NA	Neurological or psychiatric comorbidities; medications; <5 days actigraphy sleep recording	Yes	Cross-sectional	NA
Edgin, 2015 [37]	29	42 (10.3) Age range: 27–64	NA	Diagnosis of DS (including mosaicism or translocation); gestational age < 36 weeks; history of cyanotic heart defects; primary household language other than English	Yes	Cross-sectional	USA
Fernandez, 2017 [38]	66	29.86 (15.92) Age range: 5–67	NA	Gestational age < 36 weeks; <5 full consecutive days of actigraphy; child sick during recording period	Yes	Cross-sectional	USA
Hauck, 2020 [43]	9	Age range: 1–12	European American: 7 (77.8%), African American: 1 (11.1%), Hispanic/Latino: 1 (11.1%)	NA	Yes	Longitudinal	USA
Ketcheson, 2017 [40]	11	7.50 (3.14) Age range: 1–12	Caucasian: 9 (81.8%), Other: 2 (18.2%)	NA	Yes	Cross-sectional	USA
Khasgiwale, 2021 [42]	9	4.3 (0.7) Age range: 2–5	NA	Neuromuscular or neurodevelopmental diagnoses	Yes	Quasi-experimental	USA
Lloyd, 2010 [41]	30	10.7 (1.9)	Caucasian: 26 (86.7%), African American: 2 (6.7%), Other: 2 (6.6%)	Seizure disorder; noncorrectable vision problems; other medical conditions	NA	Longitudinal	USA
McKay, 2006 [30]	8	3.3 (0.3)	NA	NA	Yes	Longitudinal	USA

Note: DS = Down syndrome; TD = typical development children; NA = not available; RCT = randomized clinical trial.

**Table 4:** Technical specifications of wearable sensors.

	Actiwatch 2 ^1^	Respironics Actical ^2^	GT3X+ ^3^	Opals ^4^
Dimensions	43 × 23 × 10 mm	29 × 37 × 11 mm	33 × 46 × 15 mm	48.5 × 36.5 × 13.5 mm
Weight	16 g	16 g (without band) 22 g (with standard band)	19 g	22 g
Case material	ABS blend	Polyurethane/Polyester alloy	-	6061 clear anodized aluminum, ABS plastic
Memory size	1 Mbit	32 MB	-	8 GB
Accelerometer	Solid State Piezoelectric accelerometer. Bandwidth: 0.35–7.5 Hz. Range: 0.5–2 G peak value. Sampling rate: 32 Hz.	Range: 0.05 G to 2 G. Bandwidth: 0.035 Hz to 3.5 Hz. Sampling rate: 32 Hz.	Microelectromechanical system (MEMS)-based accelerometer and an ambient light sensor. 3-axis. Sampling rate: 30 Hz to 100 Hz.	3-axis, range: ±2 g or ±6 g. Bandwidth: 50 Hz, resolution: 14 bits. Sampling rate: 1280 Hz.
Gyroscope	Not present	Not present	Not present	3-axis. Range ±2000 º/s. Bandwidth: 50 Hz; resolution: 14 bits. Sampling rate: 1280 Hz.
Magnetometer	Not present	Not present	Not present	3-axis. Range ±6 Gauss. Bandwidth: 50 Hz; resolution: 14 bits. Sampling rate: 1280 Hz.
Logging interval	1, 15, 30 s	1, 2, 5, 15, 30, 60 s	1, 2, 3, 5, 10, 15, 30, 60, 120, 150, 180, 240 s	-
Sensitivity	0.025 G (at 2-count level)	0.02 G (at 1 G peak)	3 mg/LSB	-

Note: mm = millimeter; g = gram; MB = megabyte; GB = gigabyte; G = acceleration of gravity; Hz = hertz; °/s = degrees per second; s = second; MEMS = microelectromechanical system; ABS = acrylonitrile butadiene styrene; LSB = least significant bit; - = not reported in the manual. ^1^ = Data extracted from https://www.philips.com/c-dam/b2bhc/master/sites/actigraphy/resources/newcase/actiwatch_device_comparison.pdf. ^2^ = Data extracted from https://www.documents.philips.com/doclib/enc/9838741/Actical_Spec_Sheet.pdf. ^3^ = Data extracted from https://dl.theactigraph.com/gt3xp_wgt3xp_device_manual.pdf. ^4^ = Data extracted from https://www.mobilitysystems.se/se/wp-content/uploads/Opal_TechSpecs_APDM.pdf. All links accessed on 10 October 2020.

**Table 5:** Processing methods for wearable sensors in the included studies.

1º Author, Year	Analysis Software	Algorithm	Parameter/ Sensitivity/ Threshold	Sleep–Wake or Activity Classification Criteria
Angulo-Barroso, 2008 [44]	Mini Mitter/ Respironics software	NR	Low sensitivity (80). Threshold: 50 movement units	Sleep–wake identified within day/night blocks. Start of activity = identified when five consecutive non-zero data points were detected (corresponding to five 15 s epochs).
Arias-Trejo, 2020 [39]	Actiware 6.0	NR	Threshold: 40 counts/min for ≥5 min	Sleep periods were computed in the software and manually corrected using the sleep log.
Edgin, 2015 [37]	Actiware 5.71.0	NR	Threshold: 40 counts/min	Sleep onset = ≥3 min immobility; sleep end = ≥5 min immobility.
Fernandez, 2017 [38]	ClockLab (Actimetrics v6, Wilmette, IL, USA).	Template matching algorithm	Threshold: 40 counts/epoch	Daily onsets; daily offsets; daily acrophases.
Hauck, 2020 [43]	-	NA	-	Mean activity counts per minute over 24 h wear period.
Ketcheson, 2017 [40]	ActiLife 6	NA	NA	PA data are expressed in average counts per minute; no intensity categories defined.
Khasgiwale, 2021 [42]	Custom MATLAB analysis	NR	NR	Infant considered asleep if <3 leg movements across 5 min.
Lloyd, 2010 [41]	Mini Mitter/ Respironics software	NR	Average threshold: 131 movement units per 15 s (leg data)	Activity classified as sedentary–light (low-act) vs. moderate–vigorous (high-act), and sleep or wake state.
McKay, 2006 [30]	Mini Mitter/ Respironics software	NR	Low sensitivity (80)	Sleep and wake periods identified within day/night blocks.

Note: NR = not reported; NA = not applicable.

**Table 6:** Wearable-device-based PA and sleep protocols and findings.

1º Author, Year	Device Make/Model	Epoch	Sensor’s Units	Attachment Site	Duration	PA Findings	Sleep Findings
Angulo-Barroso, 2008 [44]	Phillips Respironics/ Actiwatch	15 s	2	Hip/Ankle	24 h	Mean counts (min), leg low act: 337.85 HI; 396.00 LG Mean counts (min), leg high act: 304.62 HI; 307.38 LG Mean counts (min), trunk low act: 268.96 HI; 304.19 LG Mean counts (min), trunk high act: 299.50 HI; 278.36 LG	NA
Arias-Trejo, 2020 [39]	Phillips Respironics/ Actiwatch 2	15 s	1	Arm	7 days	NA	Sleep efficiency (median [%]): DS: 82; TD: 89; *p* < 0.001 Sleep time (hours): DS: 8; TD: 8; *p* = 0.06 Sleep onset latency (minutes): DS: 7; TD: 6; *p* = 0.62 Fragmentation index (%): DS: 69; TD; 59; *p* = 0.04 WASO: DS: 82; TD: 52; *p* < 0.001
Edgin, 2015 [37]	Phillips Respironics/ Actiwatch 2	30 s	1	Wrist	5 days	NA	Sleep efficiency (mean [%]): DS _PS_: 74.35; DS _GS_: 83.66 TD: 85.09; *p* < 0.001 Average sleep time (minutes): DS _PS_: 460.50; DS _GS_: 509.75; TD: 511.73; *p* < 0.01 WASO (minutes) DS _PS_: 122.60; DS _GS_: 78.32; TD: 68.39; *p* < 0.001 Onset latency (minutes): DS _PS_: 9.88; DS _GS_: 10.48; TD: 13.44; *p* = 0.44 Fragmentation index (%): DS _PS_: 35.29; DS _GS_: 25.54; TD 25.50; *p* = 0.001
Fernandez, 2017 [38]	Phillips Respironics/ Actiwatch 2	30 s	1	Wrist/Ankle	7 days	NA	Sleep efficiency (average [%]): DS: 75.86; TD: 82.90 Total sleep time (minutes): DS: 453.07; TD: 489.66 Onset phase (hours): DS: 6.96; TD: 6.91 Offset phase (hours): DS: 20.87; TD: 20.86 Acrophase(hours): DS: 13.87; TD: 13.93
McKay, 2006 [30]	Phillips Respironics/ Actiwatch	15 s	1	Ankle	48 h	Integral of total activity (counts·day): 3 mo—DS: 221,733; TD: 186,932 4 mo—DS: 226,705; TD: 189,915 5 mo—DS: 207,813; TD: 232,670 6 mo—DS: 265,483; TD: 193,892 Low-Intensity activity (h·day): 3 mo—DS: 6.32; TD: 4.59 4 mo—DS: 5.93; TD: 4.99 6 mo—DS: 5.67; TD: 4.56 Time in low-intensity activity, night (min): 6 mo—DS: 76.43; TD: 35.27; *p* < 0.0125 Time in low-intensity activity integral (counts·day^−1^): 3 mo—DS: 14 975.41; TD: 10 480.61; *p* < 0.0125	Total sleep time (hours): DS: 7.30 h; TD: 9.18 h Length of night (hours): DS: 8.53 h; TD: 10.41 h Length of day (hours): DS: 15.8 h; TD: 13.51 h; *p* < 0.0125
Hauck, 2020 [43]	Phillips Respironics/ Actical	15 s	1	Ankle	24 h	CPM (counts·min): 1 mo—DS: 49.16; TD: 61.99 2 mo—DS: 56.80; TD: 85.89 3 mo—DS: 79.01; TD: 96.34 4 mo—DS: 75.29; TD: 95.79 5 mo—DS: 76.30; TD: 112.34 6 mo—DS: 55.19; TD: 122.23 12 mo—DS: 103.55; TD: 195.81 18 mo—DS: 131.48; TD: 264.54	NA
Ketcheson, 2017 [40]	Actigraph/ GT3X+	15 s	2	Wrist/Ankle	7 days	CPM (counts·min^−1^): Ankle: 1–2 mo—DS: 201.38; TD: 188.68 3–4 mo—DS: 248.28; TD: 322.01 5–6 mo—DS: 435.86; TD: 319.50 7–8 mo—DS: 433.10; TD: 397.48 9–10 mo—DS: 361.38; TD: 472.96 11–12 mo—DS: 433.10; TD: 460.38 Wrist: 1–2 mo—DS: 406.69; TD: 353.89 3–4 mo—DS: 409.36; TD: 456.07 5–6 mo—DS: 535.11; TD: 396.26 7–8 mo—DS: 481.60; TD: 633.02 9–10 mo—DS: 599.33; TD: 677.88 11–12 mo—DS: 470.90; TD: 705.30 Overall means (1–12 mo): Ankle: DS: 332.98; TD: 367.59; *p* = 0.296 Wrist: DS: 469.75; TD: 541.03; *p* = 0.171	NA
Khasgiwale, 2021 [42]	Opals, APDM/Opal	NA	2	Ankle	2 days	Post-intervention: Average leg movement rate (mov·h^1^) DS: 2350.7; TD: 3343.9; *p* = 0.002 Average leg acceleration (m·s^2^) DS: 2.09; TD: 2.34; *p* = 0.96 Peak leg acceleration (m·s^2^) DS: 4.55; TD: 4.53; *p* = 0.25 Mean movement duration (s) DS: 0.27; TD: 0.26; *p* = 0.34	NA
Lloyd, 2010 [41]	Phillips Respironics/ Actiwatch	15 s	2	Hip/Ankle	24 h	Movement/units (mean) Leg high act: 10 mo: 45,382; 12 mo: 49,446; 14 mo: 50,123 Leg low act: 10 mo: 21,810; 12 mo: 20,591; 14 mo: 23,030 Trunk high act: 10 mo: 10,296; 12 mo: 13,953; 14 mo: 16,392 Trunk low act: 10 m: 8805; 12 m: 8941; 14 m: 9212	NA

Note: PA = physical activity; HI = higher intensity, individualized treadmill training protocol; LG = lower intensity, generalized training protocol; NA = not applicable; DS = Down syndrome; TD = typical development children; WASO = wake after sleep onset; CPM = counts per minute; PS = poor sleep; GS = good sleep.

## Abbreviations

The following abbreviations are used in this manuscript:

CPM	Counts per minute
DS	Down syndrome
FAPESP	Fundação de Amparo à Pesquisa do Estado de São Paulo
GS	Good sleep
HI	Higher intensity (treadmill intervention)
JBI	Joanna Briggs Institute
LG	Lower intensity (treadmill intervention)
LMIC	Low- and middle-income countries
MeSH	Medical Subject Headings
NA	Not applicable
PA	Physical activity
PRISMA	Preferred Reporting Items for Systematic Reviews and Meta-Analyses
PROSPERO	International prospective register of systematic reviews
PS	Poor sleep
RCT	Randomized clinical trial
SB	Sedentary behavior
TD	Typical development (children)
UNIFESP	Universidade Federal de São Paulo
USA	United States of America
WASO	Wake after sleep onset
WHO	World Health Organization
